# Regional lithium prescription rates and recurrence in bipolar disorder

**DOI:** 10.1186/s40345-021-00223-7

**Published:** 2021-06-01

**Authors:** Martin Sköld, Sindre Rolstad, Erik Joas, Mathias Kardell, Erik Pålsson, Guy M. Goodwin, Mikael Landén

**Affiliations:** 1 Department of Psychiatry and Neurochemistry, Institute of Neuroscience and Physiology, Sahlgrenska Academy, University of Gothenburg, Blå Stråket 15, 413 45 Gothenburg, Sweden; 2 Department of Psychology, Faculty of Social Science, University of Gothenburg, Gothenburg, Sweden; 3 Department of Psychiatry, University of Oxford, Oxford, UK; 4 Department of Medical Epidemiology and Biostatistics, Karolinska Institutet, Stockholm, Sweden

**Keywords:** Bipolar disorder, Drug therapy, Lithium, Outcome assessment, Health care quality assessment

## Abstract

**Background:**

Lithium is the best documented maintenance treatment in bipolar disorder, but its use varies considerably across and within countries. It is not known whether regional differences in lithium prescription rates translate to differing regional outcomes.

**Aims:**

To estimate associations between county specific lithium prescription rates and county specific recurrence odds of bipolar disorder in Sweden.

**Method:**

Data from 14,616 patients with bipolar I disorder, bipolar II disorder, or bipolar disorder not otherwise specified were extracted from the Swedish national quality assurance register for bipolar disorders (BipoläR). Lithium prescription frequencies were calculated for 21 counties. Logistic regression analyses were run adjusted for confounders, with any type of recurrence as primary outcome, and incident elated and depressive episodes as secondary outcomes. Subsets of patients with bipolar I, II and not otherwise specified disorder were also analysed separately.

**Results:**

Lithium prescription rates for populations with all bipolar subtypes ranged across counties from 37.7 to 84.9% (mean 52.4%). Higher regional prescription rates were significantly associated with lower rate of any type of recurrence. The association was stronger when bipolar I disorder was analysed separately.

**Conclusions:**

The advantages for lithium use long acknowledged for bipolar I disorder are also seen for the rest of the bipolar spectrum. Results suggest that population level outcomes of bipolar disorder could be improved by increasing the number of patients using lithium.

**Supplementary Information:**

The online version contains supplementary material available at 10.1186/s40345-021-00223-7.

## Background

Bipolar disorder (BD) is a severe psychiatric disorder associated with decreased quality of life, impaired functioning, increased risk of suicide, and stigma (Guilbert 2003; Judd et al. 2005; Crump et al. 2013). The disorder is associated with high societal costs due to the need for hospital care and loss of productivity in employment (Ekman et al. 2013). The lifetime prevalence for BD has been estimated to be composed of 0.6% for bipolar I disorder (BD I), 0.4% for bipolar II disorder (BD II), and 1.4% for subthreshold bipolar spectrum disorders (Merikangas et al. 2011).

Relapse prevention is the prime objective in long term management of BD due to the high risk of recurrence of manic, hypomanic, depressive, or mixed episodes. Thus, mood stabilising medication is recommended for BD patients to prevent such episodes and in most guidelines lithium is recommended as a first-line option for maintenance treatment in adult BD (Geddes and Miklowitz 2013; Goodwin et al. 2016; Fountoulakis et al. 2016; Grunze et al. 2013; Kendall et al. 2014; Yatham et al. 2018). Swedish national recommendations derive from international guidelines and holds lithium as the first-line option for long-term treatment (Adler et al. 2014). Lithium was first shown to be effective in mania in 1949 (Cade 1949) and has been known to prevent mood episodes in BD since the 1960s (Baastrup 1964; Hartigan 1963; Ferensztajn-Rochowiak et al. 2020). A meta-analysis of randomized controlled trials (RCT) with lithium maintenance treatment (n = 1580, seven trials) demonstrated significant relative risk reductions of 34% for any type of recurrent episodes, 48% for manic episodes, and 22% for depressive episodes (Severus et al. 2014).

However, guidelines also frequently suggest other drugs as first-line options for maintenance treatment: for example, the 2018 Canadian Network for Mood and Anxiety Treatments (CANMAT) guideline endorsed by the International Society for Bipolar Disorders (Yatham et al. 2018) lists six first-line options (lithium, quetiapine, divalproex, lamotrigine, asenapine, aripiprazole) along with two combination therapies (quetiapine + lithium/divalproex, aripiprazole + lithium/divalproex). This is despite a meta-analysis indicating that lithium was superior compared with alternative medication (Miura et al. 2014) and large observational studies showing that lithium more effectively prevents recurrences and hospitalization than valproate, lamotrigine and other mood stabilizers (Joas et al. 2017; Kessing et al. 2011, 2012). Moreover, lithium seems to have a specific anti-suicidal effect (Cipriani et al. 2013) not seen with, for example, valproate (Song et al. 2017). Lastly, there is little data from RCTs on the efficacy of lithium in BD II and BD not otherwise specified (NOS) populations (Goodwin et al. 2016; Yatham et al. 2018), so there is genuine uncertainty about lithium’s value in a substantial number of patients within the bipolar spectrum.

The use of lithium has declined in Europe (Karanti et al. 2016) and the US (Blanco et al. 2002), which might be due to the regular blood-monitoring required, the awareness of adverse reactions/effects and the rise of heavily marketed alternatives. As a non-patented drug, lithium has not been marketed in a commercially conventional way. Whatever the reason, the use of lithium is often less than it should be at a time when mental health care providers are under pressure to improve outcomes by implementing evidence-based guidelines (Geddes and Miklowitz 2013; Goodwin et al. 2016; Fountoulakis et al. 2016; Grunze et al. 2013; Kendall et al. 2014; Yatham et al. 2018) and to use quality assurance programs to set goals for specific interventions. We propose that the proportion of patients treated with lithium is a potential quality indicator in BD management that is easy to measure. Quality assurance programs are concerned with populations rather than individual patients, but specifying the percentage of bipolar disorder patients that should be treated with lithium presents a challenge. This is because some individuals are likely to respond better to alternative treatment options despite lithium being the best treatment option for BD on average (Severus et al. 2014; Miura et al. 2014). Setting the goal that all BD patients should be treated with lithium would hinder personalised treatment and not yield optimal outcomes. On the other hand, if the proportion of lithium treated patients is too low, chances are that outcomes at a group level would improve by increasing the proportion of lithium treated patients.

The primary aim of this study was to investigate if outcomes in BD at a county level in Sweden are related to the prevalence of lithium prescriptions. Since it is currently not possible to set a target level for lithium use prevalence in a BD population because the optimal prevalence is unknown, the secondary aim was to investigate if a reasonable target for regional lithium prevalence can be determined.

## Methods

### Data source

Sweden has more than 100 health care quality registers that complement government-administered registries by collecting data on disease specific measures (Emilsson et al. 2015). The Swedish national quality register for bipolar disorders (BipoläR) was established in 2004 with the aim of improving the quality of care for BD patients in Sweden (BipoläR 2021). We used data from BipoläR because it contains more detailed information than the Swedish patient register where data is limited to international classification of diseases diagnoses: BipoläR contains information on, e.g., bipolar disorder subtype and psychiatric comorbidities, incident mood episodes managed in outpatient care, education, medication, and body mass index. In June 2016, the register contained more than 55,000 registrations of almost 20,000 unique patients (BipoläR 2021). Participation is voluntary for the clinician as well as the patients. Registering units include both private and public psychiatric outpatient health care units in Sweden (a total of 191 units were active in June 2016).

The quality register contains individualized data on bipolar subtype (BD I, II and NOS) and current comorbid psychiatric conditions according to Diagnostic and Statistical Manual of Mental Disorders-IV, interventions, and outcomes. The diagnoses are made in regular care and diagnostic assessments reflect clinical routine. This means that the use of structured diagnostic instruments varies across participating clinics but the mini-international neuropsychiatric interview is commonly used. During 2019, any form of structured diagnostic instrument had been used in 44% of new registrations in BipoläR. After the baseline registration, information about interventions and outcomes during the last 12 months is collected at annual follow-up examinations. At the annual follow-up, the number of depressive, manic, hypomanic, and mixed episodes are documented based on the patient interview and reviewing the past 12 months medical notes. This information is typically collected by the treating psychiatrist, or nurses trained in the diagnosis and treatment of bipolar disorder, who have access to all clinical data for the patient. Data are entered into a web-based application. Quality control is performed by data type restrictions and boundaries for registered values. Further, completeness and coverage of register data is checked annually. Diagnostic codes have also been benchmarked against medical records. Finally, the validity of the register has been assessed by comparing summary statistics to information in the national patient register and the Swedish prescribed drug register (BipoläR Årsrapport 2021).

### Study population and measures

**Fig. 1:**
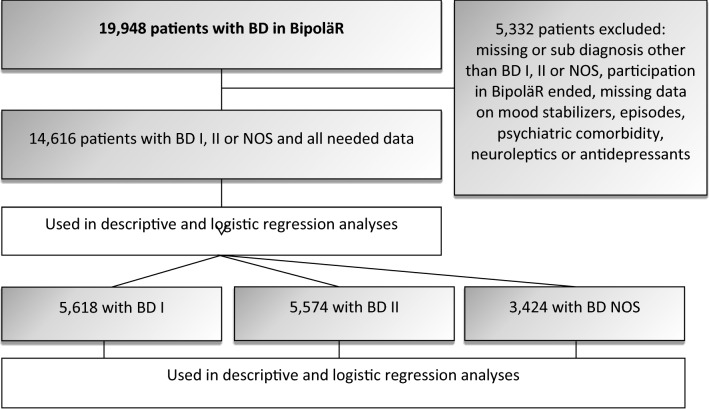
Flowchart of inclusion and exclusion of study subjects

In the current study, we included patients diagnosed with BD I, BD II, or BD NOS. At the time of data extraction (June 2016), 19,948 patients were included in BipoläR, of whom 14,616 patients were diagnosed with BD I, BD II, or BD NOS and not excluded due to other missing data (Fig. 1). Of the 14,616 patients, a total of 5618 patients were diagnosed with BD I.

The following variables were extracted from BipoläR: BD subdiagnoses, psychiatric comorbidity, number of episodes during the past 12 months, current psychotropic drug treatment, age, sex, weight, height, and educational level. We used the most recent register entry for each patient regardless of year of entry.

Dichotomous variables were created for each type of episode that had occurred during the last 12 months prior to the last register entry. Psychiatric comorbidity was extracted both as any type of comorbidity, and as the following specific comorbidities: substance abuse or addiction, anxiety disorders, and personality disorders. Mood stabilizing drugs were in this study categorized as any mood stabilizer (lithium, lamotrigine, valproate, or carbamazepine), lithium, lamotrigine, valproate, and carbamazepine. Second generation antipsychotic drugs (e.g., olanzapine and quetiapine) might also qualify as mood stabilizers (Rybakowski 2018), but were here classified as neuroleptics because they are often used temporary to treat acute episodes and as needed rescue medication.

Items included in BipoläR have been subject to change over the years, some of which affected the choice of outcomes and variables for the present analyses. For example, in 2014 the variable concerning educational level was removed. Educational level is therefore presented for all study populations along with the number of missing data, but is not included in the regression analyses.

**Fig. 2:**
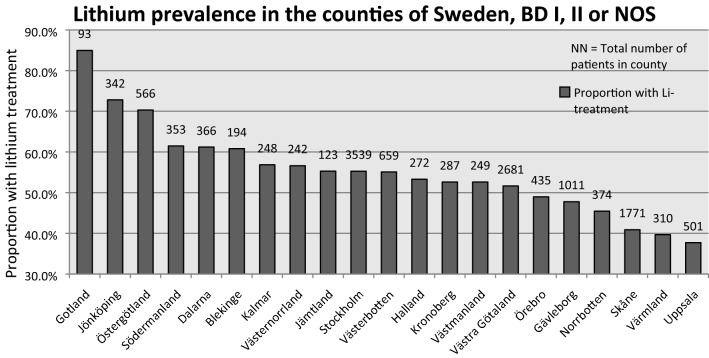
County specific lithium prevalences. All BD patients

The analyses were conducted in two steps: we first analysed the 14,616 patients with BD I, BD II, or BD NOS. Secondly, as evidence for lithium in long-term prevention varies extensively between the subtypes (strongest for BD I) (Severus et al. 2014; Miura et al. 2014), separate analyses of the subsets of patients with BD I, BD II and BD NOS were carried out. The lithium prescription rate—defined as the proportion of bipolar disorder patients being on lithium at the time for annual follow-up—was calculated for each county. The prescription rate for the total population stratified by county is shown in Fig. 2. The same calculation of prescription rate was also made for the three subpopulations BD I, BD II and BD NOS. Further details on the prevalence of lithium use in the 21 Swedish counties and for the three subsets of patients can be found in the supplement.

### Statistics

The primary outcome was any type of mood episode (coded ‘yes’ if at least one episode had occurred during the 12 months preceding the registration). Secondary outcomes were separate counts of depressive, manic, hypomanic, or mixed episode.

Logistic regression analyses conducted with relapse as the dependent variable and county lithium prevalence as the main independent variable were performed in two steps: (1) adjusted for age and sex; (2) also adjusted for psychiatric comorbidity (any type); two-sided testing was used for all analyses and the significance level was set to p < 0.05.

Results are presented with adjusted odds ratios (aOR) for a 10% point increase in lithium use prevalence, 95% confidence intervals (CI), *P*-values when applicable, and estimated (pseudo) R^2^-values (Cox–Snell and Nagelkerke) for the different county groups. No problematic multicollinearities were found for the variables age, sex, psychiatric comorbidity (any type) and lithium prescription rate. IBM SPSS version 24 was used for all analyses.

It is possible that minor changes of lithium prescription rates might have occurred within counties during the study period, meaning that the number of relapses does not perfectly match county prescription rates by year. We therefore performed a sensitivity test where we calculated county lithium prevalences for the year 2015 only, and performed regression analyses for the combined population as well as for BD I group separately. These results are found in Additional file 1.

## Results

Table 1 shows characteristics for the populations with BD I, BD II, BD NOS and the combined population. The average prescription rate of any mood stabiliser was > 80% for all the studied populations, with a 65% average lithium prescription rate in the BD I population. On average, 55% of the patients had at least one registered affective episode.

**Table 1:** Descriptive data for the study populations with BD I, BD II, BD NOS and the combined population

	BD I (n = 5618)	BD II (n = 5574)	NOS (n = 3424)	Total (n = 14,616)
Age years, mean (SD)	52.9 (15.8)	46.4 (16.0)	50.1 (16.4)	49.8 (16.3)
Gender (female), *n* (%)	3211 (57.2)	3799 (68.2)	2202 (64.3)	9212 (63.0)
Education, *n* (%)				
Less than high school	764 (13.6)	537 (9.6)	466 (13.6)	1767 (12.1)
Graduated high school	1158 (20.6)	992 (17.8)	633 (18.5)	2783 (19.0)
Graduated college or higher	1240 (22.1)	1102 (19.8)	560 (16.4)	2902 (19.9)
Missing data	2456 (43.7)	2943 (52.8)	1765 (51.5)	7164 (49.0)
BMI, mean (SD), missing	27.7 (5.4), 359	27.2 (5.7), 232	28.0 (5.7), 230	27.6 (5.6)
Psychiatric comorbidity, *n* (%)				
Any comorbidity	1137 (20.2)	1859 (33.4)	1178 (34.4)	4174 (28.6)
Substance abuse or addiction	226 (4.0)	196 (3.5)	140 (4.1)	562 (3.8)
Anxiety disorder	302 (5.4)	646 (11.6)	310 (9.1)	1258 (8.6)
Personality disorder	83 (1.5)	221 (4.0)	160 (4.7)	464 (3.2)
Mood stabilizers, *n* (%)				
Any mood stabilizers	4895 (87.1)	4610 (82.7)	2750 (80.3)	12255 (83.8)
Lithium	3664 (65.2)	2333 (41.9)	1667 (48.7)	7664 (52.4)
Lamotrigine	937 (16.7)	2242 (40.2)	924 (27.0)	4103 (28.1)
Valproate	712 (12.7)	382 (6.9)	275 (8.0)	1369 (9.4)
Carbamazepine	102 (1.8)	454 (0.8)	41 (1.2)	188 (1.3)
Neuroleptics, *n* (%)	2692 (47.9)	1837 (33.0)	1267 (37.0)	5796 (39.7)
Antidepressants, *n* (%)	1952 (34.7)	2920 (52.4)	1633 (47.7)	6505 (44.5)
Episode (at least one episode last 12 months), *n* (%), missing				
Any episode	2665 (47.4)	3614 (64.8)	1830 (53.4)	8109 (55.5)
Depressive episode	2041 (36.4), 11	3275 (58.8), 8	1603 (47.0), 12	6919 (47.4), 31
Manic episode	719 (12.9), 26	110 (2.0), 30	141 (4.1), 20	970 (6.7), 76
Hypomanic episode	1147(20.5), 20	1814 (32.6), 17	738 (21.7), 18	3699 (25.4), 55
Mixed episode	497 (8.9), 62	746 (13.4), 27	425 (12.5), 12	1668 (11.5), 101

The lithium prevalence for the combined group ranged from 37.7 to 84.9% across the 21 counties of Sweden, with an average of 52.4%. The lithium prescription rate for each county is presented in Fig. 2. The lithium prevalence for the BD I group ranged from 48.6 to 93.5% across counties, with an average of 65.2%. For the BD II and BD NOS groups, the lithium prevalence ranged from 27.0 to 77.8% (average 41.9%) and from 34.2 to 100% (average 48.7%), respectively. Percentages for each county are presented in Additional file 1.

The logistic regression analyses for the separate populations of BD I, BD II, BD NOS, as well as for the combined population are presented in Table 2. The regression analyses indicate that the odds of recurrence (any episode, depressive, and hypomanic) were higher in counties with higher lithium prevalence. This was observed at both steps of the analyses. For the aggregated population, the aOR (adjusted for age, sex and psychiatric comorbidity) for any type of episode was 0.84 (CI 0.80–0.88). The aOR can be interpreted as the decreased odds for a mood episode in the population when the lithium prescription rate is greater by 10% points. For the three subtypes, the odds ratio for relapse in any mood episode was significantly associated with lithium prevalence in all subtypes (BD I: aOR = 0.78, 95% CI 0.70–0.87; BD II: aOR = 0.90, 95% CI 0.84–0.96; BD NOS: aOR = 0.82, 95% CI 0.74–0.90).

**Table 2:** Logistic regression analyses for the study populations with BD I, BD II, BD NOS and the combined population, adjusted for age, sex, and psychiatric comorbidity

Logistic regression analyses	n	aOR	CI, 95%	*P*	*R*^*2*^
BD I					
Any episode	5618	0.78	0.70–0.87	< 0.001	0.06–0.08
Depressive episode	5607	0.81	0.72–0.90	< 0.001	0.05–0.07
Manic episode	5592	0.85	0.72–0.97	0.02	0.02–0.03
Hypomanic episode	5598	0.90	0.79–0.99	0.05	0.03–0.04
Mixed episode	5556	0.93	0.78–1.07		0.03–0.06
BD II					
Any episode	5574	0.90	0.84–0.96	0.001	0.07–0.1
Depressive episode	5566	0.93	0.87–0.99	0.02	0.06–0.08
Hypomanic episode	5557	0.87	0.80–0.93	< 0.001	0.06–0.09
Mixed episode	5547	0.96	0.87–1.04		0.03–0.06
BD NOS					
Any episode	3424	0.82	0.74–0.90	< 0.001	0.1–0.14
Depressive episode	3412	0.82	0.74–0.90	< 0.001	0.09–0.12
Hypomanic episode	3406	0.93	0.83–1.02		0.06–0.09
Mixed episode	3412	1.01	0.89–1.13		0.05–0.09
Total, all subpopulations					
Any episode	14,616	0.84	0.80–0.88	< 0.001	0.09–0.12
Depressive episode	14,585	0.87	0.83–0.92	< 0.001	0.08–0.10
Hypomanic episode	14,561	0.89	0.84–0.94	< 0.001	0.05–0.08
Mixed episode	14,515	0.94	0.88–1.01	0.097	0.04–0.07

Adjusted odds ratios (aOR) estimating differences for patients in counties with high lithium prevalence compared with counties with low lithium prevalence. The aOR can be interpreted as the decreased odds for a mood episode in the population when the lithium prescription rate is greater by 10% points

With respect to specific episodes (Table 2), higher lithium prevalence was associated with higher odds for relapse of depression in all subtypes. In BD I, higher lithium prevalence was also associated with higher odds for manic episodes. In BD II, higher lithium prevalence was also associated with higher odds for hypomanic episodes.

The results of the sensitivity analysis confined to the year 2015 corroborated the association found for the whole study period (Additional file 1).

## Discussion

There were fewer relapses in BD in Swedish counties with high lithium prescription rate than in counties with low lithium prescription rates. The association was found in the whole BD population (BD I, BD II, or BD NOS) as well as in each subpopulation of BD I, BD II and BD NOS. For BD I, a 10% point greater lithium prevalence was associated with 20% reduction in odds of relapse. Given that almost half of patients suffered at least one relapse per year, an increase of odds at this scale means that increasing lithium prescription rates on a population level has the potential to prevent a large number of relapses.

This study suggests that higher regional lithium use is associated with better regional outcomes even in a country with high average lithium use. As patients in counties with low lithium prevalence instead used other long-term medications to a greater degree, our findings also indicate that the beneficial effects of lithium cannot be fully rescued by using alternative treatments. The latter appears to conflict with the findings from a network meta-analysis by Miura et al. (2014) that suggested a general equivalence between a range of different treatments in relapse prevention studies. It should be noted, however, that almost all trials of neuroleptic drugs in BD have used an enriched RCT design that favours the study drug (Joas et al. 2017). Moreover, in RCTs typically conducted for regulatory submission, comprehensive exclusion criteria distort the study population, whereas we examined outcomes in a more heterogeneous population of BD patients.

The dataset is unusual in including BD II and BD NOS patients in substantial numbers. There are few RCTs of any plausible size that have included this population at all, especially over longer term follow-up. This is particularly true of trials that have included lithium as a treatment arm (Goodwin et al. 2016). Thus, the positive findings here are an important contribution to the existing literature and suggest that the advantages for lithium use long acknowledged for BD I patients are also seen over the rest of the bipolar spectrum.

We found no association between lithium prevalence and the recurrence rate of mixed episodes. It is possible that alternative treatments like valproate or carbamazepine have higher efficacy in atypical presentations (e.g., mixed episodes or dysphoric mania) (Cipriani et al. 2013; Fountoulakis et al. 2013). However, Joas et al. found lithium to be effective in preventing admission with a mixed episode (Joas et al. 2017). A possible explanation is that the study by Joas et al. included only hospitalizations, whereas our data also included milder relapses managed in outpatient settings. Another possible explanation is the low number of mixed episodes with ensuing low statistical power.

An intriguing corollary of these findings is that BD outcomes are likely different across countries depending on the use of lithium. Comparing outcomes in BD in relation to prescription rates of lithium across nations is challenging and requires comparable outcome data. But given the potential impact on public health, future studies should replicate these findings in the UK and other countries with data available in population. In a similar vein, the use of lithium in BD has declined in a majority of developed countries (Wolfsperger et al. 2007; Kessing et al. 2016; Karanti et al. 2016; Blanco et al. 2002; Rhee et al. 2020), even though there are examples of stable (Hayes et al. 2011) or even increasing (Castells et al. 2006) lithium prescription rates. A concerning possibility is that decreasing use of lithium has led to worse outcomes in BD. However, we are not aware of any previous study using our approach to study BD recurrence rate in relation to lithium prescription rates at a population level, let alone recurrences in relation to time trends in lithium use.

### Strengths and limitations

A strength of this study is the real-world setting and the large number of patients included: 14,616 BD patients of whom 5618 had BD I, which is much higher than for any previous RCT or even meta-analysis (Severus et al. 2014; Miura et al. 2014) and provides strong statistical power. Moreover, as already indicated, BD II and BD NOS are rarely even included in clinical trials but are represented at an appropriate level here.

Among limitations to consider are first that the data source for the current study, BipoläR, only covers approximately 30% of the total Swedish bipolar population (BipoläR 2021). However, general trends observed in BipoläR, including a higher prevalence of bipolar disorder in women and secular trends in mood stabilizer treatment, are mirrored in the Swedish patient and prescribed drugs registers. This supports the assumption that observations from BipoläR are applicable to the total Swedish bipolar population. The study population was narrowed further mainly due to missing data. Even though the patients included in the current study thus comprise approximately 22% of the total Swedish bipolar population, there is a risk of selection bias. It is possible that the study has oversampled patients from well-functioning care providers as registering patients in BipoläR might be an indication of better care. Second, mood episodes were identified by clinicians who assessed the course during the last 12 months. In particular depressive episodes might be difficult to delineate as subsyndromal symptoms might linger for long time. Third, there are many other factors associated with specific counties that might impact outcomes, for instance psychiatric health care organization and culture, economic resources, and societal factors like unemployment rate, or educational level. Unfortunately, we were unable to control for potential differences in educational levels across the populations due to a change in the quality register BipoläR where this variable was removed. It is possible that a more well-functioning psychiatric health care system is the cause of both higher lithium prescription and less recurrences. Future studies should take into account other factors potentially reflecting quality of care that might confound the results. Fourth, patients who had changed their medical treatment during the last year were not excluded because the exact date when the change occurred is not known. This imposes a risk that recurrent episodes or their absence are a result of an older treatment regime rather than the current treatment. However, the latter can be seen as added noise that is more likely to diminish positive associations than to boost them. Fifth, we used data from patients’ last registration during the study period 2004–2016. Even though the great majority of patient data were from the end of the study period and peaked during 2015, small changes of lithium prescription rates within counties during the study period means that the number of relapses does not perfectly match county prescription rates by year. We therefore conducted sensitivity analyses for year 2015 only, which corroborated the associations found for the whole period; see Additional file 1.

## Conclusions

We provide evidence of an association between higher lithium prevalence rate and lower recurrence of illness at a regional level. Although no causal inference can be made directly from this study, it is compatible with results from prospective clinical trials and suggests that population outcomes across the bipolar spectrum (BD I, BD II, and BD NOS) might be improved by increasing the number of patients treated with lithium. Lithium prescription rate might hence serve as a quality indicator of BD care. Indeed, outcomes continued to improve with higher lithium prevalence despite the mean lithium prevalence for the population being 65.2% (BD I), which is much higher than in most other countries. Higher levels than this would be a worthwhile target in the quest to improve management of bipolar patients.

## Supplementary Information

**Additional file1: Table S1.** County specific lithium prevalences. All BD patients. **Table S2.** County specific lithium prevalences for BD I, BD II, and BD NOS. **Table S3.** Logistic regression analyses for patients with BD I, BD II, or BD NOS only year 2015. OR estimating differences for patients in counties with high lithium prevalence compared to counties with low lithium prevalence. Adjusted for age, sex and psychiatric comorbidity. **Table S4.** Logistic regression analyses for patients with BD I only year 2015. OR estimating differences for patients in counties with high lithium prevalence compared to counties with low lithium prevalence. Adjusted for age, sex and psychiatric comorbidity. **Figure S1.** Inclusion and exclusion flow chart.

## Data Availability

The authors had full and ongoing access to the original data presented and analysed in this study. Due to Swedish legal restrictions, register data cannot be shared. However, the data that support the findings of this study are available from the corresponding author upon reasonable request.

## Abbreviations

aOR: Adjusted odds ratio
BD: Bipolar disorder
BDI: Bipolar I disorder
BD II: Bipolar II disorder
BipoläR: The Swedish national quality register for bipolar disorders
CANMAT: Canadian network for mood and anxiety treatments
NOS: Not otherwise specified
RCT: Randomized controlled trial
