# Supplementary material for: Regional lithium prescription rates and recurrence in bipolar disorder
Source: Int J Bipolar Disord. 2021 Jun 1;9:18. doi: 10.1186/s40345-021-00223-7 (PMC8167923; doi:10.1186/s40345-021-00223-7)
Supplement: Supplementary file 1 — Additional file1: Table S1. County specific lithium prevalences. All BD patients. Table S2. County specific lithium prevalences for BD I, BD II, and BD NOS. Table S3. Logistic regression analyses for patients with BD I, BD II, or BD NOS only year 2015. OR estimating differences for patients in counties with high lithium prevalence compared to counties with low lithium prevalence. Adjusted for age, sex and psychiatric comorbidity. Table S4. Logistic regression analyses for patients with BD I only year 2015. OR estimating differences for patients in counties with high lithium prevalence compared to counties with low lithium prevalence. Adjusted for age, sex and psychiatric comorbidity. Figure S1. Inclusion and exclusion flow chart. [file 40345_2021_223_MOESM1_ESM.docx]

# SUPPLEMENTARY Material

Supplementary Table 1: County specific lithium prevalences. All BD patients.

| County | Lithium treatment | | Total no. of patients in county |
| --- | --- | --- | --- |
|  | n | % |  |
| Gotland | 79 | 84.90 | 93 |
| Jönköping | 249 | 72.80 | 342 |
| Östergötland | 398 | 70.30 | 566 |
| Södermanland | 217 | 61.50 | 353 |
| Dalarna | 224 | 61.20 | 366 |
| Blekinge | 118 | 60.80 | 194 |
| Kalmar | 141 | 56.90 | 248 |
| Västernorrland | 137 | 56.60 | 242 |
| Stockholm | 1956 | 55.30 | 3539 |
| Jämtland | 68 | 55.30 | 123 |
| Västerbotten | 363 | 55.10 | 659 |
| Halland | 145 | 53.30 | 272 |
| Kronoberg | 151 | 52.60 | 287 |
| Västmanland | 131 | 52.60 | 249 |
| Västra Götaland | 1385 | 51.70 | 2681 |
| Örebro | 213 | 49.00 | 435 |
| Gävleborg | 483 | 47.80 | 1011 |
| Norrbotten | 170 | 45.50 | 374 |
| Skåne | 724 | 40.90 | 1771 |
| Värmland | 123 | 39.70 | 310 |
| Uppsala | 189 | 37.70 | 501 |
| Total | 7664 | 52.40 | 14616 |

Supplementary Table 2: County specific lithium prevalences for BD I, BD II, and BD NOS.

| BDI | | | |  | BDII | | | |  | NOS | | | |
| --- | --- | --- | --- | --- | --- | --- | --- | --- | --- | --- | --- | --- | --- |
| County | Lithium treatment | | Total no of patients |  | County | Lithium treatment | | Total no of patients |  | County | Lithium treatment | | Total no of patients |
|  | n | % |  |  |  | n | % |  |  |  | n | % |  |
| Gotland | 29 | 93.5 | 31 |  | Gotland | 42 | 77.8 | 54 |  | Gotland | 8 | 100.0 | 8 |
| Östergötland | 206 | 82.1 | 251 |  | Jönköping | 107 | 77.0 | 139 |  | Östergötland | 104 | 64.6 | 161 |
| Dalarna | 89 | 76.7 | 116 |  | Östergötland | 88 | 57.1 | 154 |  | Jämtland | 14 | 63.6 | 22 |
| Jönköping | 112 | 73.2 | 153 |  | Blekinge | 41 | 55.4 | 74 |  | Södermanland | 75 | 63.6 | 118 |
| Södermanland | 78 | 72.2 | 108 |  | Jämtland | 28 | 50.9 | 55 |  | Kalmar | 48 | 61.5 | 78 |
| Blekinge | 55 | 71.4 | 77 |  | Dalarna | 62 | 50.8 | 122 |  | Västernorrland | 24 | 61.5 | 39 |
| Västmanland | 58 | 69.0 | 84 |  | Södermanland | 64 | 50.4 | 127 |  | Jönköping | 30 | 60.0 | 50 |
| Norrbotten | 78 | 68.4 | 114 |  | Västernorrland | 49 | 49.5 | 99 |  | Dalarna | 73 | 57.0 | 128 |
| Halland | 55 | 67.1 | 82 |  | Halland | 47 | 48.5 | 97 |  | Västerbotten | 154 | 53.8 | 286 |
| Västerbotten | 121 | 66.9 | 181 |  | Västmanland | 34 | 45.9 | 74 |  | Kronoberg | 50 | 53.2 | 94 |
| Stockholm | 1103 | 66.1 | 1668 |  | Västerbotten | 88 | 45.8 | 192 |  | Gävleborg | 132 | 52.6 | 251 |
| Kalmar | 61 | 65.6 | 93 |  | Västra Götaland | 453 | 42.8 | 1058 |  | Stockholm | 317 | 51.4 | 617 |
| Västra Götaland | 707 | 64.6 | 1094 |  | Stockholm | 536 | 42.7 | 1254 |  | Blekinge | 22 | 51.2 | 43 |
| Kronoberg | 62 | 64.6 | 96 |  | Örebro | 61 | 41.8 | 146 |  | Halland | 43 | 46.2 | 93 |
| Gävleborg | 183 | 62.5 | 293 |  | Kalmar | 32 | 41.6 | 77 |  | Värmland | 57 | 45.2 | 126 |
| Västernorrland | 64 | 61.5 | 104 |  | Kronoberg | 39 | 40.2 | 97 |  | Västmanland | 39 | 42.9 | 91 |
| Skåne | 326 | 58.1 | 561 |  | Norrbotten | 53 | 36.3 | 146 |  | Västra Götaland | 225 | 42.5 | 529 |
| Örebro | 119 | 58.0 | 205 |  | Gävleborg | 168 | 36.0 | 467 |  | Örebro | 33 | 39.3 | 84 |
| Jämtland | 26 | 56.5 | 46 |  | Skåne | 256 | 30.9 | 828 |  | Skåne | 142 | 37.2 | 382 |
| Uppsala | 96 | 51.3 | 187 |  | Värmland | 30 | 27.3 | 110 |  | Uppsala | 38 | 34.5 | 110 |
| Värmland | 36 | 48.6 | 74 |  | Uppsala | 55 | 27.0 | 204 |  | Norrbotten | 39 | 34.2 | 114 |
| Total | 3664 | 65.2 | 5618 |  | Total | 2333 | 41.9 | 5574 |  | Total | 1667 | 48.7 | 3424 |

Supplementary Figure 1. Inclusion and exclusion flow chart

19,948 patients with BD in BipoläR

18,613 with BD type I, II or NOS

Excluded:
765 sub diagnose missing
500 schizoaffective syndrome
70 cyclothymic

17,842 with lithium data and still in register

Excluded:
97 lithium data missing
674 patient participation ended (all reasons)

15,344 with relapse data

Excluded:
2,498 recurrence data missing

15,113 with mood stabilizer data

14,768 with psychiatric comorbidity data

Excluded:
231 mood stabilizer data last 12 months missing

Excluded:
345 psychiatric comorbidity missing

Excluded:
152 neuroleptics and/or antidepressants data missing

5,618 with BD I

Used in descriptive analyses and logistic regression analyses

14,616 with psychiatric comorbidity data

Used in descriptive analyses and logistic regression analyses

3424 with BD NOS

5574 with BD II

Supplementary Table 3: Logistic regression analyses for patients with BD I, BD II, or BD NOS only year 2015. OR estimating differences for patients in counties with high lithium prevalence compared to counties with low lithium prevalence. Adjusted for age, sex and psychiatric comorbidity.

| Logistic analyses | OR | CI, 95% | p | *R^2^* |
| --- | --- | --- | --- | --- |
| Any episode, *n=8024* | 0.85 | 0.79 - 0.91 | <0.001 | 0.09 - 0.12 |
| Depressive episode, *n=8007* | 0.90 | 0.85 - 0.96 | 0.001 | 0.08 - 0.11 |
| Manic episode, *n=7971* | 0.91 | 0.80 - 1.03 | 0.13 | 0.004 - 0.01 |
| Hypomanic episode, *n=7991* | 0.94 | 0.88 - 1.00 | 0.07 | 0.06 - 0.09 |
| Mixed episode, *n=7961* | 0.93 | 0.85 - 1.02 | 0.13 | 0.04 - 0.08 |

Supplementary Table 4: Logistic regression analyses for patients with BD I only year 2015. OR estimating differences for patients in counties with high lithium prevalence compared to counties with low lithium prevalence. Adjusted for age, sex and psychiatric comorbidity.

| Logistic analyses | OR | CI, 95% | p | *R^2^* |
| --- | --- | --- | --- | --- |
| Any episode, *n=2942* | 0.75 | 0.66 - 0.84 | <0.001 | 0.07 - 0.09 |
| Depressive episode, *n=2934* | 0.82 | 0.72 - 0.91 | <0.001 | 0.06 - 0.08 |
| Manic episode, *n=2925* | 0.83 | 0.70 - 0.97 | 0.02 | 0.02 - 0.04 |
| Hypomanic episode, *n=2930* | 0.87 | 0.76 - 0.98 | 0.02 | 0.03 - 0.05 |
| Mixed episode, *n=2618* | 0.87 | 0.72 - 1.03 | 0.1 | 0.04 - 0.08 |
